# Race and ethnicity moderate the associations between lifetime psilocybin use and past year hypertension

**DOI:** 10.3389/fpsyt.2024.1169686

**Published:** 2024-06-24

**Authors:** Grant M. Jones, Jocelyn A. Ricard, Matthew K. Nock

**Affiliations:** ^1^ Department of Psychology, Harvard University, Cambridge, MA, United States; ^2^ Department of Neuroscience, Stanford University, Stanford, CA, United States

**Keywords:** race, psilocybin, NSDUH, hypertension, psychedelic

## Abstract

**Background:**

Hypertension is a major source of morbidity and mortality worldwide, particularly for racial and ethnic minorities who face higher rates of hypertension and worse health-related outcomes. Recent research has reported on protective associations between classic psychedelics and hypertension; however, there is a need to explore how race and ethnicity may moderate such associations.

**Methods:**

We used data from the National Survey on Drug Use and Health (2005–2014) to assess whether race and ethnicity moderate the associations between classic psychedelic use – specifically psilocybin – and past year hypertension.

**Results:**

Hispanic identity moderated the associations between psilocybin use and past year hypertension. Furthermore, individuals who used psilocybin and identified as Non-Hispanic White had reduced odds of hypertension (aOR: 0.83); however, these associations were not observed for any other racial or ethnic groups in our study for individuals who used psilocybin.

**Conclusion:**

Overall, our results demonstrate that the associations between psychedelics and hypertension may vary by race and ethnicity. Longitudinal studies and clinical trials can further advance this research and determine whether such differences exist in causal contexts.

**Project registration:**

https://osf.io/xsz2p/?view_only=0bf7b56749034c18abb2a3f8d3d4bc0b.

## Introduction

Hypertension is a major worldwide health concern that leads to increased risk of cardiovascular disease and premature death (1, 2). The use of classic psychedelics – naturally-occurring or derived serotonin 2A receptor agonists that can elicit non-ordinary states of consciousness (3) – has been identified as a potential protective factor for hypertension (4); although, direct investigations of this matter have never been conducted and thus this link remains an open question for future research. Examples of classic psychedelics include psilocybin (the active compound in “magic mushrooms”), lysergic acid diethylamide (LSD), and peyote (a cactus with psychoactive properties).

Despite the possible protective associations between classic psychedelic use and hypertension, there is a need to explore if race and ethnicity might affect these associations for two reasons. First, research on classic psychedelics has suffered from a lack of inclusion and focus on racial and ethnic minorities, limiting the generalizability of prior findings suggesting that these substances may share protective associations with hypertension. Second, there are long documented differences in risk levels for hypertension across different races and ethnicities that may affect the associations that psychedelics share with hypertension as well (5–7). Additionally, there are well-known health disparities in hypertension outcomes for racial and ethnic minorities (8, 9) that may also cause associations between psychedelic use and hypertension to differ for these populations. Thus, the goal of this investigation is to examine if race and ethnicity moderate the associations between classic psychedelic use and hypertension.

Although there is preliminary evidence that classic psychedelics may share protective associations with hypertension, this research suffers from a lack of focus on racial and ethnic minorities and is preliminary. First, Simonsson and colleagues used data from the National Survey on Drug Use and Health (NSDUH) (10) and found protective associations between lifetime classic psychedelic use and hypertension, even after controlling for a host of other potential confounds (e.g., demographic factors, lifetime use of other substances, etc.) (4). Although this work laid an essential foundation for better understanding the link between classic psychedelics and hypertension, this research also did not examine how race and ethnicity may moderate these associations, inviting further investigation into this research domain. Additionally, as psychedelics can also cause transient increases in blood pressure, cardiovascular disease is an exclusion criteria for many psychedelic trials (11); relatedly, there have been no in-depth clinical investigations into the impact of psychedelic use on hypertension. Thus, there is generally a need for more research within this domain, including how race/ethnicity might play a role in the link between psychedelics and hypertension.

Second, healthy lifestyle changes can have a considerable impact on hypertension outcomes (12, 13) and comprehensive reviews have described a number of studies that indicate classic psychedelics can promote such changes (14). However, there is a dearth of research on whether race and ethnicity might affect potential lifestyle changes associated with classic psychedelics. For example, in a study with over 340 participants who stopped or reduced alcohol consumption or misuse after a psychedelic experience, over 60% of the individuals reported improved diet and over 50% reported increased exercise as a result of their experience with psychedelics (15). Yet, nearly 90% of participants in this study were White and only 5.8% were Hispanic, sharply limiting the generalizability of these results. In a similar vein, a study by Anderson et al. (16) found that spontaneous health behavior changes such as improved eating habits and increased exercise were associated with microdosing classic psychedelics. Although these researchers recruited from over a dozen countries, they nevertheless acknowledge that their sample was predominantly “middle class, white, male, and heterosexual”, illustrating that the lack of racial diversity in psychedelic research is pervasive and exists even when one samples within different cultural contexts.

Third, although there is evidence that classic psychedelics may treat mental health disorders that confer increased risk of hypertension, this research has suffered from a lack of diversity as well (17). Classic psychedelics have demonstrated efficacy for treating internalizing disorders such as anxiety and depression – disorders that are known risk factors for hypertension (18, 19) – in a number of high-impact clinical trials (20–23). However, in a comprehensive review of foundational psychedelic trials that have been conducted in recent years, over 80% of participants in these trials were White whereas under 5% of participants were Black or Hispanic (24). Amidst this lack of diversity, there is a clear need to investigate whether prior results on psychedelics and adverse mental health outcomes that put one at risk for hypertension might generalize to diverse populations.

Furthermore, there is preliminary evidence suggesting that associations between psychedelics and adverse health outcomes may differ for racial and ethnic minorities (25, 26), inviting further inquiries into whether such differences exist for associations involving hypertension.

Using data from the National Survey of Drug Use and Health (NSDUH), Jones and Nock (26) and Jones (25) demonstrated that race and ethnicity significantly moderate the associations that psychedelics share with psychological distress, suicidality, and depression. For White participants, both MDMA/ecstasy and psilocybin conferred decreased odds of the aforementioned outcomes. However, for racial and ethnic minorities, there were far fewer of such associations (25, 26). For White participants in Jones & Nock (26), MDMA/ecstasy and psilocybin shared lowered odds of distress and suicidality, but for Black, Hispanic, and Multiracial participants, these associations were non-existent. Additionally, in Jones (25) and for White participants, psilocybin once again conferred lowered odds of depression but did not for Black and Multiracial participants; additionally, for Hispanic participants, psilocybin and MDMA/ecstasy were associated with lowered odds of just one of three depression outcomes, and for Indigenous participants, MDMA/ecstasy was associated with increased odds of depression for two of three outcomes. Additionally, Jones et al. (27) found that psilocybin conferred lower odds of crime arrests for all racial and ethnic groups aside from Black and Hispanic individuals. These results have important implications for psychedelic research as they suggest limits to the generality of existing findings on psychedelics.

Thus, in light of the above findings, the goal of this study is to examine whether race and ethnicity moderate the associations between classic psychedelics and hypertension in a population-based survey sample. This investigation is also a follow-up study to Simonsson et al. (4), which previously found protective associations between classic psychedelics and hypertension (4). Although our investigation cannot be used to make causal claims about the impact of race and ethnicity on the effects of psychedelics, it can make important advancements in our understanding of the associations among identity, psychedelic use, and health.

## Methods

Data are from the National Survey on Drug Use and Health (2005–2014), an annual survey that aims to collect information on drug use and health outcomes in a nationally-representative sample of the U.S. population. The NSDUH recruits participants by reaching out to a randomly selected, nationally-representative assortment of household addresses across the United States, and collects data via a computer-assisted self-interviewing paradigm wherein field-interviewers administer the survey for participants to complete in their homes. The NSDUH collects data from individuals aged 12 years and older across all 50 states and the District of Columbia. Additionally, the NSDUH survey does not collect data from individuals without a fixed household address, such as active duty military members and homeless individuals who do not reside in shelters. We used data from 2005–2014 as these are all of the years in the NSDUH survey for which there are data on our dependent variable of interest (self-reported hypertension). Lastly, we included all participants aged 18+ years and older in the current study.

This study was exempt from Harvard Institutional Review Board (IRB) review as all data for this project are publicly available at the following web address: https://www.datafiles.samhsa.gov/data-sources.

### Analysis plan

Our analysis plan consists of three main steps. For each step, we conducted survey weighted logistic regression models, allowing us to incorporate the survey design and weighting from the NSDUH into our analyses.

**Step 1.** First, we assessed the associations that each of the four individual classic psychedelics (psilocybin, peyote, mescaline, and LSD) shared with past-year hypertension. Given that Simonsson et al. found lifetime tryptamine use (i.e., psilocybin) in particular conferred lowered odds of hypertension, we hypothesized that psilocybin would be the substance that conferred lowered odds of hypertension in this follow-up study. This hypothesis was further driven by prior cross-sectional population-based studies demonstrating that tryptamines share the strongest protective associations with health outcomes (e.g., distress, suicidality) compared with other types of psychedelics (28), suggesting that we might see similar results in the current study.

**Step 2.** After identifying the substance(s) that conferred lowered odds of hypertension, we planned to test whether race and ethnicity significantly moderate the associations that psychedelics share with hypertension.

**Putative Moderator.** In line with prior research in this domain (25, 26), we used a three level race and ethnicity variable for our moderation analyses (Non-Hispanic White [*N =* 242,527], Non-Hispanic Participant of Color [*N =* 79,677], Hispanic [*N =* 59,478]), reduced from the seven level race/ethnicity variable included in the NSDUH (Non-Hispanic White, Black [*N =* 46,896], Native American/Alaska Native [N = 5,702], Native Pacific Islander/Hawaiian [*N =* 1,923], Asian [*N =* 14,688], Multiracial [*N =* 10,468], Hispanic). We reduced the number of levels of our moderator *a priori* for statistical parsimony and to reduce the number of interaction tests in our study, ultimately reducing the likelihood of a Type 1 error (false positive). In interpreting our interactions, we were not concerned with the specific beta values yielded by this model, but simply whether any of the tests were significant, as any positive test would serve to justify the next step of our study.

**Step 3.** Finally, if our moderation tests yielded any significant results, we planned to break down our sample by race and ethnicity and assess associations between relevant psychedelics and hypertension for individual racial and ethnic groups. This approach allows us to incorporate the unique demographic and substance use profiles of each demographic group into our analyses and generate specific assessments of how the protective associations between psychedelics and hypertension vary across groups.

**Dependent Variable (yes/no).** Self-reported hypertension in the past year served as the dependent variable for our analyses. Additionally, one’s self-report of hypertension was based on whether a medical professional had diagnosed a given participant with this condition in the past year.

**Independent Variable (yes/no).** Lifetime use of any of four classic psychedelics (psilocybin, peyote, mescaline, and LSD) served as the independent variable for our study.

**Covariates.** In line with our prior population based survey research on race and psychedelics (25, 26), we used the following covariates in our analyses: sex, age, income, educational attainment, marital status, survey year, and lifetime use of various substances (MDMA/ecstasy, PCP, inhalants, cocaine, heroin, tranquilizers, stimulants, sedatives, pain relievers, and marijuana).

## Results

The demographics of our sample, broken down by race and ethnicity, are presented in **Table 1**. Rates of hypertension were significantly lower amongst Hispanic participants than they were among Non-Hispanic White and Non-Hispanic Participants of Color. In contrast, rates of classic psychedelic use were higher among Non-Hispanic White participants than among the other racial and ethnic groups in the study.

**Table 1:** Sample demographics and prevalence of past year hypertension and past year substance use, broken down by race and ethnicity.

Characteristic	(Non-Hispanic) White (weighted %) (N = 242,527)	(Non-Hispanic) Participant of Color (weighted %) (N = 79,677)	Hispanic (weighted %) (N = 59,478)	p-value^1^
Past Year Hypertension	20.2%	21.2%	10.3%	<0.001
Marital Status				<0.001
Married	57.7%	41.8%	50.6%	
Widowed	6.8%	5.5%	3.5%	
Divorced or Separated	13.4%	15.3%	12.6%	
Never Been Married	22.0%	37.3%	33.3%	
Educational Attainment				<0.001
Less than HS	2.6%	3.5%	18.8%	
Some HS/HS Grad	38.8%	41.8%	45.7%	
Some College or Above	58.6%	54.7%	35.5%	
Age				<0.001
18–25	12.9%	17.5%	20.2%	
26–34	13.7%	18.2%	22.9%	
35–49	26.3%	29.3%	31.2%	
50+	47.1%	35.0%	25.7%	
Sex				<0.001
Male	48.4%	45.6%	50.7%	
Female	51.6%	54.4%	49.3%	
Yearly Household Income				<0.001
<$20,000	14.5%	27.3%	26.4%	
$20,000-$49,999	30.9%	33.5%	41.9%	
$50,000-$74,999	18.8%	14.9%	13.9%	
$75,000+	35.9%	24.2%	17.8%	
Self Reported Engagement in Risky Behavior				<0.001
Never	45.4%	64.1%	65.7%	
Seldom	38.8%	25.0%	22.9%	
Sometimes	14.5%	9.7%	9.8%	
Always	1.3%	1.1%	1.5%	
Lifetime Psilocybin Use	11.4%	3.1%	4.9%	<0.001
Lifetime LSD Use	12.8%	4.3%	5.5%	<0.001
Lifetime Peyote Use	2.9%	1.3%	1.2%	<0.001
Lifetime Mescaline Use	4.5%	1.7%	1.5%	<0.001
Lifetime MDMA/Ecstasy Use	6.9%	4.4%	5.1%	<0.001
Lifetime Cocaine Use	18.1%	10.3%	13.0%	<0.001
Lifetime Heroin Use	1.9%	1.6%	1.4%	<0.001
Lifetime PCP Use	3.1%	2.1%	1.9%	<0.001
Lifetime Inhalant Use	10.5%	4.0%	6.1%	<0.001
Lifetime Pain Reliever Use	15.8%	10.3%	12.0%	<0.001
Lifetime Tranquilizer Use	11.3%	4.9%	6.2%	<0.001
Lifetime Stimulant Use	10.3%	3.8%	5.0%	<0.001
Lifetime Sedative Use	4.4%	1.6%	1.8%	<0.001
Lifetime Marijuana Use	48.9%	38.1%	32.1%	<0.001

^1^chi-squared test with Rao & Scott’s second-order correction.

The results of our preliminary assessment of the associations between the four most commonly used classic psychedelics (psilocybin, peyote, mescaline, and LSD) are presented in **Table 2**. In line with our hypothesis, psilocybin was the sole classic psychedelic substance that conferred lowered odds of hypertension among the four we assessed. Thus, lifetime psilocybin use served as the independent variable within our analyses.

**Table 2:** Associations between lifetime use of individual classic psychedelics and past year hypertension.

Lifetime Use	aOR (95% CI)^1^	p-value
Psilocybin	0.82*** (0.74, 0.91)	<0.001
LSD	0.94 (0.86, 1.04)	0.224
Peyote	0.90 (0.79, 1.02)	0.090
Mescaline	0.97 (0.88, 1.08)	0.609

^1^*p<0.05; **p<0.01; ***p<0.001; aOR, adjusted odds ratio; CI, confidence interval.

The results of our interaction tests are presented in **Table 3**. Overall, the “Hispanic” category interacted significantly with psilocybin for predicting past year hypertension. Given that one of our interaction tests was significant, we proceeded with breaking down our sample by race and ethnicity and assessing the associations that psilocybin shared with hypertension for each racial and ethnic group.

**Table 3:** Interaction test between race and ethnicity and lifetime psilocybin use for predicting past year hypertension.

Lifetime Use	beta (95% CI)^1^	p-value
Race/Ethnicity * Psilocybin		
(Non-Hispanic) Participant of Color	0.04 (-0.21, 0.30)	0.731
Hispanic	0.48** (0.12, 0.84)	0.009

^1^*p<0.05; **p<0.01; ***p<0.001; CI, confidence interval.

The associations between classic psychedelics and hypertension, broken down by race and ethnicity, are presented in **Table 4**. Psilocybin conferred lowered odds of hypertension only for Non-Hispanic White participants (aOR: 0.83; 95% CI [0.75, 0.92]). There were no other significant associations between psilocybin and hypertension.

**Table 4:** Associations between lifetime psilocybin use and past year hypertension, broken down by race and ethnicity.

Group	aOR (95% CI)^1^	p-value
White	0.83*** (0.75, 0.92)	<0.001
Black	0.91 (0.58, 1.43)	0.693
Indigenous	0.55 (0.29, 1.03)	0.061
Asian	0.78 (0.29, 2.05)	0.605
Multiracial	0.93 (0.50, 1.70)	0.804
Hispanic	0.82 (0.56, 1.21)	0.322

^1^*p<0.05; **p<0.01; ***p<0.001; aOR, adjusted odds ratio; CI, confidence interval.

## Limitations

This study features a number of limitations that are important to state clearly. First and foremost, our study is based on cross-sectional data, meaning that our findings cannot be used to make definitive causal claims. Future longitudinal studies and clinical trials will be needed to address these limitations and determine whether there are causal links between race and ethnicity, psychedelic use, and hypertension.

Second, there are limitations due to the large sample size in this study and our ability to detect small effects as significant. The significant odds ratios in this study are not much lower than 1 (with an OR of 1 representing an independent variable having no significant association with a given dependent variable), suggesting that the associations between psilocybin use and hypertension may be minimal. Future replication studies can address this limitation and determine whether the aforementioned associations exist within other samples.

Third, due to the nature of our main independent variable – lifetime psilocybin use – we could not assess a number of important dosing parameters within this study, such as frequency, dosage, and recency of use. Future studies that include more granular measures of psychedelic use will be needed to overcome this limitation.

Fourth, given that the NSDUH is based on self-report, underreporting represents another potential limitation. This limitation is especially relevant for this study as racial and ethnic minorities are at increased risk of criminal repercussions due to illicit substance use, given the racialized nature of the criminal justice system (29). If racial and ethnic minorities report psychedelic use less frequently than Non-Hispanic White participants, this underreporting may potentially contribute to differences in the associations between psilocybin and hypertension. Follow-up work can assess whether there are differences in the response rates for questions on illegal substances, and subsequently test whether these differences may impact the associations between psychedelics and adverse outcomes.

Fifth, this study used broad, imprecise categories to assess racial and ethnic identity (White, Black, Indigenous, Asian, Multiracial, and Hispanic). Furthermore, the “Hispanic” category has particular limitations, given that this is an ethnic category that includes individuals with a diverse range of racial identities. These limitations stem from the seven-level race/ethnicity variable included in the NSDUH (Non-Hispanic White, Non-Hispanic Black, Non-Hispanic Asian, Non-Hispanic Native American, Non-Hispanic Native Hawaiian/Pacific Islander, Non-Hispanic Multiracial, and Hispanic), which limits our ability to assess identity in more granular ways in this study. There are more comprehensive and flexible ways to assess race and ethnicity in future work (e.g., more detailed race/ethnicity variables, qualitative assessments, assessments of perceived racial identity, etc.). Additionally, it is also likely that a complex interplay of identity factors (i.e., gender, immigration status, socioeconomic status) impact the associations between psychedelic use and hypertension as well. Future investigations should therefore take a more nuanced approach to studying the impact of identity on psychedelics and health, as such investigations can shed further light on our observed findings.

Finally, another potential limitation is that the interaction test for Non-Hispanic racial and ethnic minorities was not significant and the confidence intervals for White participants and some racial and ethnic minority groups overlap, calling into question the validity of our observed differences. However, we do not believe this limitation to hinder our findings. Given that our interaction tests involve pooling the variance from White and Non-White participants to conduct a test of differences, one is disproportionately weighting the model terms of the interaction around White participants. Such a test therefore represents a stringent assessment of differences, meaning that one null result should not prevent further exploration of potential differences between groups using complementary statistical methods. Additionally, our study featured a robust number of participants of color, instilling confidence that our models yielded accurate estimates of the associations between psilocybin and hypertension for each racial and ethnic group. Nevertheless, future longitudinal research and studies featuring even larger sample sizes can address this limitation.

## Discussion

We found that race and ethnicity significantly moderate the previously observed protective association between classic psychedelics – specifically, psilocybin – and hypertension. More specifically, this protective association existed only for Non-Hispanic White participants. These results extend prior research examining the associations between classic psychedelics and hypertension (4), while also replicating recent findings demonstrating fewer protective associations between psychedelics and adverse outcomes for those from racial and ethnic minority groups (25, 26).

### Potential explanations for the observed findings

Despite the above limitations, our study makes a significant contribution to the literature by demonstrating the associations between psychedelic use and hypertension vary by racial and ethnic identity. Furthermore, we believe there are a few potential explanations for our results.

**Third Variable Factors.** Third variable factors may underlie the observed results, as prior research suggests there may be important pre-drug differences between those who have versus have not used psychedelics that may contribute to the associations between these substances and health outcomes. For instance, Johnstad (30) analyzed a sample of 319 individuals who use psychedelics and found that they scored significantly higher on each of the Big Five personality traits except for extraversion (agreeableness, openness, conscientiousness, and neuroticism) (30). Furthermore, a 2017 study by Nour, Evans, and Carhart-Harris found that lifetime psychedelic use was positively associated with a number of potential pre-drug factors, such as affinity with nature, openness, and liberal political views (31). Lastly, there is also evidence that personality factors contribute to a diagnosis of hypertension as well (32, 33), lending further credence to third variable factors as an explanation of our findings. Future investigations can explore a range of third-variable factors as potential mediators of the observed moderation effect in this study.

Additionally, in our study there may be additional demographic differences that exist within and between racial groups that contribute to the observed findings. For example, rates of hypertension are known to increase with age (34), and in our study, there is a higher proportion of 50+ Non-Hispanic White participants (~48% of White participants are aged 50+) than there are for Hispanic participants (~28% of Hispanic participants are aged 50+). Although we controlled for age in all of our analyses, there may be third variable demographic differences that we did not control for that may have driven these results. Other potential third-variable demographic factors that may have driven our results include parental education, level of religiosity, rural vs. urban status, and household ownership status; these factors remain purely speculative, however. Future research can explore how additional third variable demographic factors impact the associations between psilocybin, race, and hypertension.

**Racial disparities in hypertension.** Second, there are many disparities in hypertension outcomes that exist for communities of color; downstream, such disparities may contribute to our findings in this study demonstrating fewer protective associations between psilocybin use and hypertension. Existing research indicates that racial and ethnic minorities – especially Black Americans and Hispanic Americans – have poorer hypertension outcomes across a host of dimensions (8, 9). Compared to Non-Hispanic White individuals, Hispanic adults have also been noted to have lower levels of hypertension awareness and blood pressure control (35–37). Furthermore, the prevalence of hypertension, as well as hypertension-related deaths, is also elevated for American Indians as compared to many other racial and ethnic groups (38, 39). A 2011–2012 study additionally noted Non-Hispanic Asian individuals had lower awareness of their hypertensive status as well as lower treatment rates for hypertension than did adults from other racial and ethnic groups (39, 40). Lastly, there are generally pervasive disparities in access to healthcare treatment for communities of color (41) which may exacerbate differences in hypertension outcomes for communities of color compared to White populations.

If psilocybin use does share a direct or indirect relationship to reductions in hypertension, such disparities may weaken protective associations that may exist in this domain. Although there is suggestive evidence of potential causes that may underlie these disparities (e.g., differing treatment effects across racial groups, body-mass index differences, dietary factors), no single factor has been identified to explain differences in hypertension outcomes for racial and ethnic minorities (42). Therefore, future research should further explore the causes and conditions that give rise to hypertension disparities, as well as the potential biological mechanisms that may link psilocybin use to lowered odds of hypertension, as these inquiries may shed further light on our observed findings.

### Potential health implications of our results

While we cannot make definitive causal claims based on the results in this study, they nevertheless may have important implications for population health and future clinical investigations in this research area.

The odds ratios (ORs) being lower than 1.0 for the relationship between psilocybin use and hypertension in this study indicates that there may be a protective effect of naturalistic psilocybin use on hypertension. It is important to note that effects in our study were small, with the significant ORs ranging between 0.82–0.83 and the non-significant ORs ranging between 0.55–0.93; these ORs are within the range of those observed within prior work in this area, which found ORs of 0.80–0.86 for the associations between psilocybin use and hypertension (4). Despite the small effects, these results still may have important implications for health if they are causal. Foundational health research indicates that small effect sizes can have a large impact in key contexts (43), such as when small effects are applied to large groups. Accordingly, as this study is a large-scale investigation featuring over 380,000 participants, these small effect sizes may indicate a slight, diffuse protective effect of psilocybin use on hypertension at the population-level. However, further research is needed to substantiate this claim.

Furthermore, the significant moderation effect of race/ethnicity on the link between psilocybin and hypertension may have important implications for clinical research as well. If these results apply to causal and experimental contexts, it may imply that psilocybin may vary in its efficacy as a treatment for hypertension depending on one’s racial or ethnic background. Such interpretations are tentative and much further research is needed to validate this possibility. Yet, the clear results in this study – whereby a protective association between psilocybin use and hypertension existed only for Non-Hispanic White participants – certainly invite further inquiry.

### A “call to action” for future research

Therefore, we recommend that these findings serve as a “call to action” that motivates additional inquiries into the link between race/ethnicity, psychedelic use, and hypertension. Specifically, we recommend four areas of future research. First, we recommend additional cross-sectional studies, using databases like the NSDUH, to better understand the epidemiology of psychedelic use and hypertension for racial and ethnic minority communities. For instance, future analyses can assess trends in psychedelic use for minority communities and see if trends may differ for those with versus without hypertension. Such studies can start to shed light on the differential patterns of psychedelic use within diverse populations and provide additional context for the results in this study. Second, future qualitative work is essential as well. Qualitative work on the intersection of race/ethnicity, hypertension, and psychedelic use may provide invaluable information about the lived experiences of individuals of color who use psychedelics, and why such lived experiences may have resulted in the differential findings between populations of color versus White populations in the observed work. Third, we recommend longitudinal studies as well. Future studies can recruit large, diverse samples of psychedelic users and matched controls, and document how hypertension changes over time for populations of color compared to White populations. Such findings would enable future causal investigations of our results. And finally, clinical trials can provide definitive answers about whether psychedelics like psilocybin may hold potential for alleviating hypertension and whether these effects may differ for different communities.

## Conclusion

This study assessed the moderating role of race and ethnicity on the protective associations that classic psychedelics – specifically psilocybin – share with hypertension. Protective associations between psilocybin use and hypertension existed only for Non-Hispanic White participants. These findings may be explained by third variable factors or racial and ethnic disparities in hypertension outcomes. Additional cross-sectional and qualitative studies can provide more context for our results; furthermore, longitudinal and clinical studies also represent a propitious next step in this line of research, as such studies can assess whether these findings are causal. Overall, this study advances our understanding of the impact that identity may have on the associations between psychedelic use and health, and tees up future research in this domain of inquiry.

## Data availability statement

Publicly available datasets were analyzed in this study. This data can be found here: https://www.samhsa.gov/data/data-we-collect/nsduh-national-survey-drug-use-and-health.

## Ethics statement

The requirement of ethical approval was waived by Harvard University Institutional Review Board for the studies involving humans because the data are publicly available. The studies were conducted in accordance with the local legislation and institutional requirements. Written informed consent for participation was not required from the participants or the participants’ legal guardians/next of kin because the data are publicly available.

## Author contributions

GJ: Conceptualization, Formal analysis, Writing – original draft. JR: Writing – original draft, Writing – review & editing. MN: Writing – original draft, Writing – review & editing.
